# Pupil size as an indicator of cognitive activity in mild Alzheimer’s disease

**DOI:** 10.17179/excli2021-4568

**Published:** 2022-01-17

**Authors:** Mohamad El Haj, Guillaume Chapelet, Ahmed A. Moustafa, Claire Boutoleau-Bretonnière

**Affiliations:** 1 Nantes Université, Univ. Angers, Laboratoire de Psychologie des Pays de la Loire (LPPL - EA 4638), F-44000 Nantes, France; 2 Unité de Gériatrie, Centre Hospitalier de Tourcoing, Tourcoing, France; 3 Institut Universitaire de France, Paris, France; 4 CHU Nantes, Clinical Gerontology Department, Bd Jacques Monod, F44093, Nantes, France; 5 Université de Nantes, Inserm, TENS, The Enteric Nervous System in Gut and Brain Diseases, IMAD, Nantes, France; 6 School of Psychology, Faculty of Society and Design, Bond University, Gold Coast, Queensland, Australia; 7 Department of Human Anatomy and Physiology, Faculty of Health Sciences, University of Johannesburg, Johannesburg, South Africa; 8 CHU Nantes, Inserm CIC04, Département de Neurologie, Centre Mémoire de Ressources et Recherche, Nantes, France

**Keywords:** Alzheimer's disease, memory, pupil, pupillometry, working memory span tasks

## Abstract

It is well established that pupil activity indexes cognitive processing. For instance, research has consistently demonstrated that the pupil reacts to working memory span task performance. However, little is known about pupil reaction to cognitive processing in Alzheimer's Disease (AD). We thus investigated whether span tasks can modulate pupil size in patients with AD. We invited 24 patients with AD and 24 healthy older adults to perform backward and forward spans, as well as to count aloud in a control condition, while their pupil activity was recorded with eye tracking glasses. In patients with AD, analysis demonstrated larger pupil size during backward spans (*M* = 2.12, *SD* = .39) than during forward spans (*M* = 1.98, *SD* = .36) [*t*(23) = 3.22, *p *= .004], larger pupil size during forward spans than during counting (*M* = 1.67, *SD* = .33) [*t*(23) = 4.75, *p *< .001], as well as larger pupil size during backward spans than during counting [*t*(23) = 10.60, *p *< .001]. In control participants, analysis demonstrated larger pupil size during backward spans (*M* = 3.36, *SD* = .49) than during forward spans (*M* = 2.85, *SD* = .68) [*t*(23) = 5.82, *p *< .001], larger pupil size during forward spans than during counting (*M* = 2.09, *SD* = .62) [*t*(23) = 5.42, < .001], as well as larger pupil size during backward spans than during counting [*t*(23) = 9.70, *p *< .001]. Results also demonstrated a significant interaction effect between groups and conditions [*F*(2,92) = 16.63, *p *< .001]; in other words, patients with AD have shown fewer variations on the pupil size across the conditions compared to the control participants. The larger pupil size during backward spans, compared with forward spans or counting, can be attributed to the high cognitive load of backward spans. The modulation of pupil size, as observed across backward/forward spans and counting, can possibly be attributed to sympathetic/adrenergic and parasympathetic/cholinergic activities. Our study demonstrates the value of pupillometry as a potential biomarker of cognitive processing in AD.

## Introduction

Alzheimer's Disease is a neurological disorder characterized by the deposition of amyloid (Aβ) plaques and intracellular neurofibrillary tangles, along with neuronal degeneration in the brain (McKhann et al., 2011[29]). These neural deteriorations are associated with the main cognitive hallmark of AD, namely, with a substantial decline of memory (McKhann et al., 2011[29]). In clinical situations, the evaluation of memory decline in AD is typically based on paper-and-pencil tasks. On these tasks, patients are typically invited to verbally repeat a series of single digits in the same order (i.e., forward span) or in the reverse order (i.e., backward span) or even to retain a word-list for a later recall test. While these paper-and-pencil tasks are well-validated, they do not offer physiological indexes of memory dysfunction in AD. It is timely to provide a validated physiologically-based evaluation of memory in AD. Such evaluation may contribute to precision medicine, that is, individually-tailored diagnosis (Hampel et al., 2019[18]). Our paper addresses this point by assessing whether memory performances in patients with AD can be physiologically indexed with pupil size. In this study, we highlight basic proprieties of pupil functioning, as well as research demonstrating how pupil reacts to working memory span tasks. We also highlight research demonstrating how variations in pupil size can reflect neural mechanisms of memory decline in AD.

The pupil, the opening area of the iris that allows light to reach the retina, is controlled by the sphincter muscles that decrease the diameter of pupil and dilator muscles that increase its diameter (Kawasaki, 1999[23]; Sirois and Brisson, 2014[38]). The pupil sphincter and dilator muscles, respectively, receive impulses from the parasympathetic (i.e., cholinergic) and sympathetic (i.e., adrenergic) autonomic nervous systems (Kawasaki, 1999[23]). In other words, the pupil size is controlled by the balance of two antagonistic muscles: the sphincter, which reacts to activation of the parasympathetic system, and the dilator, which reacts to activation of the sympathetic stimuli. The pupil sphincter and dilator muscles serve to optimize vision by modulating the amount of light that reaches the retina; whereas the pupil constricts in brighter conditions it dilates in darker conditions (Kawasaki, 1999[23]). 

Besides its reaction to light (Fotiou et al., 2007[12]), pupil size reacts to memory function (Goldinger and Papesh, 2012[15]; Kucewicz et al., 2018[25]; Bergt et al., 2018[4]). In a seminal work, Kahneman and Beatty (1966[21]) evaluated pupil dilation during working memory span task performance in young healthy participants. Results demonstrated increased pupil dilation in response to increased difficulty of the tasks. Similar findings were reported by subsequent studies demonstrating that pupil dilation increases with each digit retained in digit span tasks until the length of the digits exceeds the capacity of working memory, at which pupil diameter begins to plateau or diminish (Peavler, 1974[31]; Granholm et al., 1996[17]; Cabestrero et al., 2009[5]; Wahn et al., 2016[39]; Alnaes et al., 2014[2]). This body of research demonstrates that pupil dilation can be a reliable and valid physiological marker of cognitive load, that is, the measurement of pupil dilation can provide an online indication of the amount of cognitive effort devoted to working memory (Laeng et al., 2012[26]). A similar suggestion was made by research on pupil dilation during the retrieval of personal information. One study has investigated pupil dilation during the retrieval of personal information and during a control condition in which participants had to count aloud (El Haj et al., 2019[7]). Results demonstrated larger pupil diameters during the retrieval of personal information than during the control task. Further, the increased pupil size was attributed to the cognitive load related to the retrieval of personal information. 

Variations in pupil size during span tasks may also reflect neural processes in AD. As mentioned above, the pupil sphincter muscle receives impulses from the parasympathetic (i.e., cholinergic) autonomic nervous systems (Kawasaki, 1999[23]). More specifically, the pupil sphincter reacts to acetylcholine which is a neurotransmitter involved in projections between the Edinger-Westphal nucleus, ciliary ganglion, and sphincter muscle (Kawasaki, 1999[23]). Research has demonstrated that AD patients have reduced levels of acetylcholine (ACh), resulting in increased pupillary size and changes in the pupillary responses including reduced latency and amplitude of pupillary light reflex (Shen and Wu, 2015[36]; Singh and Verma, 2020[37]; Ornek et al., 2015[30]). The root cause of cholinergic deficiency in AD is the degenerative pathological change in the Edinger-Westphal nucleus and nucleus basalis of Meynert (Singh and Verma, 2020[37]). Research has even shown that the Edinger-Westphal nucleus is affected at early stages of AD, displaying deposition of Aβ amyloid plaques and neurofibrillary tangles (Scinto et al., 1999[35], 2001[34]). Interestingly, a significant correlation has been reported between Aβ and tau levels in cerebrospinal fluid and pupil size in AD (Frost et al., 2013[14]), which may make pupillometry a potential biomarker for AD. Thus, pupillometry may provide a practical and noninvasive evaluation of neural mechanisms in patients with AD. 

To summarize, the pupil is intimately connected to the physiological and chemical processes of the brain, including memory function. The link to memory processes has been demonstrated by a large body of research demonstrating increased pupil dilation in response to increased difficulty of working memory span tasks (Kahneman and Beatty, 1966[21]; Peavler, 1974[31]; Granholm et al., 1996[16]; Cabestrero et al., 2009[5]; Wahn et al., 2016[39]; Alnaes et al., 2014[2]). However, this research has not investigated pupil size changes in AD. The current study thus investigates whether span tasks can modulate pupil size in patients with AD. We thus invited patients with AD and healthy older adults to perform backward and forward span tasks, as well as to count aloud in a control condition, while their pupil activity was recorded with an eye tracking glasses. We expected larger pupil size during backward spans, compared with forward spans or counting, which may be attributed to the high cognitive load of backward spans.

## Method

### Participants

The study included 24 patients with a clinical diagnosis of probable AD (14 women, *M *age in years = 72.33, *SD* = 6.33, *M* education in years = 8.00, *SD* = 2.17) and 24 healthy older adults (13 women, *M *age in years = 70.96, *SD* = 6.34, *M* education in years = 8.29, *SD* = 2.07). The diagnosis of amnestic form of AD dementia was made by an experienced neurologist or geriatrician based on the criteria of the National Institute on Aging-Alzheimer's Association (McKhann et al., 2011[29]). The patients were in the mild stages of AD (*M *Mini Mental State Examination = 22.58, *SD* = 1.79) and were recruited from memory clinics. Regarding control participants, they were independent and living at home and were matched with the AD patients according to gender [*X*^2^(1, *N *= 48) = .85,* p *= .36], age [*t*(46) = .75, *p *= .46], and educational level [*t*(46) = .48, *p* = .64]. The control group has demonstrated higher general cognitive ability [*M *Mini Mental State Examination = 27.92, *SD* = .88, *t*(46) = 13.08, *p *< .001] compared to the patients with AD.

Exclusion criteria for all participants were significant psychiatric or neurological illnesses, alcohol or drug use, or a history of clinical depression. No participants presented any major visual or auditory acuity difficulties that would have prevented completion of the study tasks. Patients who are administered drugs (e.g., tropicamide) that could alter pupillary dilatation were also excluded. The sample size was determined a priori using G*Power and calculation was conducted for 2 groups X 3 conditions ANOVA repeated measures (see the statistical analysis section). Based on 95 % power, an estimated probability of making Type I error of .05, and an effect size of .25, the calculation suggested a total sample size of 44 participants. We, however, included four additional participants to increase the statistical power. The study was conducted in accordance with the principles of the Declaration of Helsinki with a favorable opinion (number 20202-A02276-33) from the Committee for the Protection of Persons (the French national ethical board).

## Procedures and Materials

Participants were tested in two span conditions (i.e., forward and backward) as well as in a control condition (i.e., counting). We implemented the control condition to compare pupil size during spans vs. a condition not requiring working memory. The three conditions were counterbalanced across participants and, during each condition, participants wore eye-tracking glasses and faced a white wall. Participants were tested individually in a quiet room at the memory clinic for patients with AD and an experimental box at the university of Nantes for control participants. To ensure that differences in pupil dilation were not caused by differences in retinal illumination, blinds were closed and the lightness of the room (60-watt fluorescent lamp) was the same for all participants and across the three conditions. Prior to the experiment, participants were informed that the experiment was related to eye tracking research and cognition in general. In order not to influence their performance, the participants were not provided with further details regarding pupil dilation and its relationship to cognitive performance. 

In the span tasks, we applied procedures from the WAIS-R (Wechsler, 1981[40]). In the WAIS-R, the reliability coefficient of spans was .87, and their exploratory factor relatively to working memory was .67. We invited participants to repeat aloud sequences of digits of increasing length read out by the experimenter, either in the same order (i.e., forward spans) or in the reverse order (i.e., backward spans). The experimenter pronounced the digits aloud and the frequency of pronunciation was one number per sec. Two trials of each sequence length were administered. The forward sequences began with three single digits up to the maximum capacity of participants. The backward sequences also began with three digits up to the maximum capacity of participants. The procedure was stopped if the participants made an error in two consecutive trials of the same length. Performance on the forward and backward spans referred to the number of digits in the correctly repeated last sequence. In the counting control condition, we invited participants to count aloud up from one during two minutes. We implemented this control condition because it requires, like the span tasks, counting aloud involves verbal behavior, so any potential differences in pupil dilation between the counting and spans is not the result of verbal behavior. 

Spans and counting occurred while pupil in the dominant eye was recorded using the Pupil Capture software. Participants wore eye-tracking glasses (Pupil Lab) consisting of a remote pupil-tracking system that uses infrared illumination with 200 Hz sampling rate and a gaze position accuracy of < 0.1° prior to each trial (i.e., counting, forward and backward spans). We recorded data from the dominant eye and calibration was made by inviting participants to fixate on a black cross (a 5 x 5 cm cross, printed on an A4 white paper fixated at the wall center) that was used as a calibration reference; the cross was withdrawn after calibration. During spans and counting, participants were seated in front of a white wall and the distance between the participants and wall was approximately 30-50 cm. Participants were instructed not to look outside the wall, but were free to explore all parts of it. The wall did not display any visual stimuli (e.g., drawings, windows). Regarding dependent variables, we calculated the mean of pupil dilation (in mm) during the two span conditions as well as the mean of pupil dilation during counting. During data analysis, links were identified by the typical loss of corneal reflection and were automatically excluded from the data.

### Statistical analysis

We compared pupil size using repeated measures ANOVA with the two groups (i.e., AD vs controls) as the between-factor and the three conditions (i.e., counting, forward and backward spans) as the repeated measures, followed by Bonferroni correction. Note that the pupil size data were plotted and checked for normal distribution with Kolmogorov-Smirnov tests. The normality tests have, however, demonstrated that the spans' data were not normally distributed. Accordingly, we used for the spans' data Mann-Whitney U-test for inter-group comparisons, and Wilcoxon's signed-rank test for within-group comparisons. For all tests, the level of significance was set as *p* ≤ 0.05.

## Results

### Increased pupil size on backward spans

**Figure 1:**
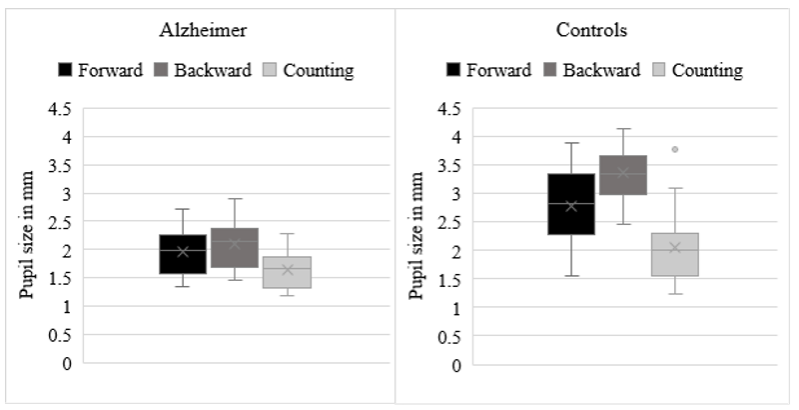
Means of pupil diameters during the forward and backward spans and during the control condition (i.e., counting) in patients with Alzheimer's disease and control participants

Means of pupil diameters are depicted in Figure 1(Fig. 1). Analyses showed a significant group effect [*F*(1, 46) = 50.51, *p* < .001, η2 = .5], indicating smaller pupil size in AD patients (*M* = 1.92, *SD* = .36) than in control participants (*M* = 2.77, *SD* = .55). The condition effect was also significant [*F*(2,92) = 37.57, *p* < .001, η2 = .61]. Follow-up paired *t*-tests demonstrated, in all participants overall, larger pupil size during backward spans (*M* = 2.74, *SD* = .76) than during forward spans (*M* = 2.42, *SD* = .70) [*t*(46) = 5.97, *p *< .001], larger pupil size during forward spans than during counting (*M* = 1.88, *SD* = .54) [*t*(46) = 6.17, *p *< .001], as well as larger pupil size during backward spans than during counting [*t*(46) = 8.60, *p *< .001]. The interaction effect between group and condition was also significant, *F*(2,92) = 16.63, *p *< .001, η2 = .27. Independent samples-*t* tests demonstrated smaller pupil size in AD patients than in control participants on the backward spans [*M* AD = 2.12, *SD* = .39, *M* controls = 3.36, *SD* = .49, *t*(46) = 9.71, *p* < .001], forward spans [*M* AD = 1.98, *SD* = .36, *M* controls = 2.85, *SD* = .68, *t*(46) = 4.45, *p* < .001], and counting [*M* AD = 1.67, *SD* = .33, *M* controls = 2.09, *SD* = .62, *t*(46) = 2.89, *p* = .006]. In AD patients, paired-*t* tests demonstrated larger pupil size during backward spans than during forward spans [*t*(23) = 3.22, *p *= .004], larger pupil size during forward spans than during counting [*t*(23) = 4.75, *p *< .001], as well as larger pupil size during backward spans than during counting [*t*(23) = 10.60, *p *< .001]. In control participants, paired-*t* tests demonstrated larger pupil size during backward spans than during forward spans [*t*(23) = 5.82, *p *< .001], larger pupil size during forward spans than during counting [*t*(23) = 5.42, < .001], as well as larger pupil size during backward spans than during counting [*t*(23) = 9.70, *p *< .001].

### Decreased spans in AD

Regarding performances on spans, analysis demonstrated lower forward spans in AD patients (*M* = 4.50, *Median* = 4.00, *SD *= 1.10) than in control participants (*M* = 5.67, *Median* = 6.00, *SD *= .92) (*Z* = 3.61, *p *< .001, Cohen's *d* = 1.22), as well as lower backward spans in AD patients (*M* = 3.54, *Median* = 3.50, *SD *= 1.11) than in control participants (*M* = 4.50, *Median* = 5.00, *SD *= .59) (*Z* = 3.86, *p* < .001, Cohen's *d* = 3.17). Analysis also demonstrated lower forward than backward spans in AD patients (*Z* = 3.03, *p* = .002, Cohen's *d* = 1.55) and control participants (*Z* = 3.76, *p* < .001, Cohen's *d* = 2.39).

### Complementary analysis

For convenience, we conducted correlational analyses between pupil size and performances on the span tasks. Analysis demonstrated significant correlations between forward spans and pupil size on these spans in AD patients (*r* = .48, *p* = .018) and control participants (*r* = .58, *p* = .003). Analysis also demonstrated significant correlations between backward spans and pupil size on these spans in AD patients (*r* = .49, *p* = .015) and control participants (*r* = .57, *p* = .004). 

## Discussion

Our results demonstrate that performing backward spans results in a larger pupil size compared to forward spans, and that both forward and backward spans result in a larger pupil size compared with counting. These results demonstrate that pupil size can mirror cognitive processing in AD and healthy older adults.

The larger pupil size during backward than forward spans, as well as during forward spans than during counting, can be attributed to cognitive load. As discussed above, Kahneman and Beatty (1966[21]) have demonstrated how increasing cognitive load, by increasing the spans to be remembered, increased pupillary size. The relationship between cognitive load and pupil size was earlier demonstrated by Hess and Polt (1964[19]) who demonstrated that increasing cognitive load, by increasing the level of multiplication operations, increased pupillary size. Subsequent research has provided further evidence on the relationship between pupillary dilation and cognitive load (Peavler, 1974[31]; Granholm et al., 1996[16]; Cabestrero et al., 2009[5]; Wahn et al., 2016[39]; Alnaes et al., 2014[2]; Ahern and Beatty, 1979[1]; Karatekin et al., 2004[22]; Piquado et al., 2010[32]). This research supports the assumption that pupil size can indicate the intensity of cognitive load (Just and Carpenter, 1993[20]). This cognitive load assumption fits with our procedures because backward spans require extensive cognitive processing (i.e., the activation of the executive system in working memory to manipulate and reproduce the sequence in the reversed order) in comparison to forward spans that involve the activation of automatic processing of the slave systems in working memory to immediate recall without manipulation of the spans (Baddeley, 1992[3]). Furthermore, compared to forward spans, counting involves less cognitive load (i.e., simply ascribing a point value to the number “one” and so on). Together, the larger pupil size during backward than during forward spans, as well as during forward spans than during counting, as observed in our study, can be attributed to the increasing cognitive load of the tasks. In other words, pupil size can be used as an index of cognitive load in AD. 

The cognitive load hypothesis can be further supported by a study assessing the ability of patients with AD to detect changes across conditions involving processing of colors and shapes that differed across the trials (Fernández and Parra, 2021[11]). Results demonstrated less pupil dilation during the encoding of the differed stimuli than during retrieval. According to this study, the altered pupil dilation can be attributed to inefficient encoding mechanisms in the patients. While this study has also suggested relationship between pupil variations and cognitive load, the procedures have not included a task involving increased cognitive load in the span tasks. The span tasks were, however, assessed in a study linking pupil dilation to the functioning of the *locus coeruleus* in AD (Elman et al., 2017[8]). While this study has demonstrated changes in pupil dilation across different load levels, only forward spans were used, which is different from our study in which we have assessed and compared forward span, backward span, and control task performance. The same argument can be made regarding a study demonstrating increased pupil size in patients with AD on a forward span task (Granholm et al., 2017[17]). 

The cognitive load hypothesis is also supported by study on normal aging. Piquado et al. (2010[32]) monitored pupil size in older adults and younger adults while processing digit lists that varied in length, this to observe increased pupil size with the length of digits in both populations. These findings demonstrate how the pupil size increases with the increased cognitive load in normal aging, which mirrors findings in the current study.

Besides the cognitive load account, pupil size as observed in AD patients can potentially be attributed to activities in the sympathetic (i.e., adrenergic) and parasympathetic (i.e., cholinergic) autonomic nervous systems. Pupil dilation involves activation of preganglionic sympathetic neurons, as well as by inhibitory regulation of the Edinger-Westphal nucleus (Kawasaki, 1999[23]). Thus, the larger pupil size during backward than forward span task or counting, as observed in our study, can be arguably attributed to a higher activation of preganglionic sympathetic neurons as well as to higher inhibitory activity in the Edinger-Westphal nucleus. Conversely, the smaller pupil size during counting than during forward or backward spans can mirror higher activity in Edinger-Westphal nucleus and, more specifically, to higher levels of acetylcholine, resulting in a higher activity in the sphincter muscles and, consequently, the smaller pupil size during counting. The variations in pupil size across our three experimental conditions can thus potentially mirror both adrenergic and cholinergic activities. This issue is important because AD has been associated with deficits in both adrenergic (Kelly et al., 2017[24]; Prettyman et al., 1997[33]) and cholinergic activities (Shen and Wu, 2015[36]; Singh and Verma, 2020[37]; Ornek et al., 2015[30]; Scinto et al., 2001[34]). 

Our study demonstrates the value of pupillometry as a potential biomarker of cognitive processing in AD. Research has typically evaluated pupil activity in AD regarding pupillary light response; for a comprehensive review, see Chougule et al. (2019[6]). Few studies have evaluated the value of pupillometry, and retinal activity in general (Frost et al., 2013[13]; Feke et al., 2015[9]; Fernández et al., 2016[10]; Lim et al., 2016[27]; Marandi and Gazerani, 2019[28]), as a potential biomarker in AD. One study has, however, found significant correlations between pupil size and Aβ and tau levels in cerebrospinal fluid in AD (Frost et al., 2013[14]). That being said, no published research has evaluated the value of pupillometry as a potential biomarker of cognitive processing in AD. Our study tackles this challenge by demonstrating how pupil size can variate following the cognitive activity as processed by patients with AD. Further, as demonstrated by the significant interaction effect between groups and conditions, patients with AD have shown fewer variations on the pupil size across the conditions compared to the control participants, which may be considered as the key finding of our study. This interaction effect was mirrored by the correlation analysis, demonstrating reduced correlations, at least numerically, between pupil size and spans in AD patients compared to controls. This interaction effect demonstrates that AD patients have not only an overall reduced pupil response compared to controls, but also a much smaller variation as a function of cognitive load. We believe that these findings (i.e., the fewer pupil variations in AD patients compared with healthy older adults) provide useful information for the diagnosis of AD as pupil responses in AD seem to differ from normal aging, at least for our study design. We believe that our study paves the way to the design and use of pupillometry as a practical and noninvasive biomarker of working memory, and cognition in general, in AD. This biomarker may significantly contribute to advancing precision medicine that seeks to implement key technological and scientific breakthrough, especially in neurosciences, to offer individually-tailored diagnosis (Hampel et al., 2019[18]).

One may argue that our study design may lack the sophistication and refinement provided by computer-based paradigms (e.g., a design in which participants rehearse information presented on a screen). While the latter paradigm may offer a precise measurement of pupil activity during the perception of a given stimulus, we have designed our study to offer an ecological procedure that may be easily implemented in memory clinics. Furthermore, if our design has involved the processing of outer stimuli, the pupil activity would be attributed to by the characteristics (or the presentation-speed) rather than to its cognitive processing per se.

To summarize, as “the window to the brain,” the eye provides substantial information to the brain. As proposed by our study, this window, through pupil size, can offer a valuable biomarker of cognitive processing in AD. Our study paves the way to the use of pupillometry, as a practical and non-invasive biomarker, to improve the diagnosis of cognitive decline in AD. On the long term, this biomarker can be used in addition to the available *in vivo*-biomarkers as proposed for an early identification of AD pathology and cognitive decline, including brain imaging biomarkers (e.g., magnetic resonance imaging, positron emission tomography) and fluid biomarkers (e.g., cerebrospinal fluid, blood-based biomarkers). 

## Declaration

### Authorship contribution 

MEH: Conceptualization, methodology, assessment, writing - original draft, software, formal analysis. GC: Analysis, interpretation, review & editing. AAM and GC: Analysis, interpretation, review & editing. CBB: Resources, investigation, supervision, interpretation, review & editing.

### Funding

The first author was partially supported by the project Etoile Montante (Région Pays de la Loire).

### Institutional review board statement

The study was approved by the Committee for the Protection of Persons (20202-A02276-33).

### Informed consent statement

Informed consent was obtained from all subjects involved in the study.

### Data availability statement

The datasets used and/or analyzed during this study is available as electronic supplement.

### Conflict of interest

The authors declare that they have no conflict of interest.

## Supplementary Material

Supplementary data

## References

[1] Ahern S, Beatty J (1979). Pupillary responses during information processing vary with Scholastic Aptitude Test scores. Science.

[2] Alnaes D, Sneve MH, Espeseth T, Endestad T, van de Pavert SH, Laeng B (2014). Pupil size signals mental effort deployed during multiple object tracking and predicts brain activity in the dorsal attention network and the locus coeruleus. J Vision.

[3] Baddeley A (1992). Working memory. Science.

[4] Bergt A, Urai AE, Donner TH, Schwabe L (2018). Reading memory formation from the eyes. Eur J Neurosci.

[5] Cabestrero R, Crespo A, Quirós P (2009). Pupillary dilation as an index of task demands. Percept Mot Skills.

[6] Chougule PS, Najjar RP, Finkelstein MT, Kandiah N, Milea D (2019). Light-induced pupillary responses in Alzheimer's disease. Front Neurol.

[7] El Haj M, Janssen SMJ, Gallouj K, Lenoble Q (2019). Autobiographical memory increases pupil dilation. Transl Neurosci.

[8] Elman JA, Panizzon MS, Hagler DJ, Eyler LT, Granholm EL, Fennema-Notestine C (2017). Task-evoked pupil dilation and BOLD variance as indicators of locus coeruleus dysfunction. Cortex.

[9] Feke GT, Hyman BT, Stern RA, Pasquale LR (2015). Retinal blood flow in mild cognitive impairment and Alzheimer's disease. Alzheimers Dement (Amst).

[10] Fernández G, Manes F, Politi LE, Orozco D, Schumacher M, Castro L (2016). Patients with mild Alzheimer’s disease fail when using their working memory: Evidence from the eye tracking technique. J Alzheimer's Dis.

[11] Fernández G, Parra MA (2021). Oculomotor behaviors and integrative memory functions in the Alzheimer’s clinical syndrome. J Alzheimer's Dis.

[12] Fotiou DF, Brozou CG, Haidich A-B, Tsiptsios D, Nakou M, Kabitsi A (2007). Pupil reaction to light in Alzheimer’s disease: evaluation of pupil size changes and mobility. Aging Clin Exp Res.

[13] Frost S, Kanagasingam Y, Sohrabi H, Vignarajan J, Bourgeat P, Salvado O (2013). Retinal vascular biomarkers for early detection and monitoring of Alzheimer's disease. Transl Psychiatry.

[14] Frost S, Kanagasingam Y, Sohrabi HR, Taddei K, Bateman R, Morris J (2013). Pupil response biomarkers distinguish amyloid precursor protein mutation carriers from non-carriers. Curr Alzheimer Res.

[15] Goldinger SD, Papesh MH (2012). Pupil dilation reflects the creation and retrieval of memories. Curr Dir Psychol Sci.

[16] Granholm E, Asarnow RF, Sarkin AJ, Dykes KL (1996). Pupillary responses index cognitive resource limitations. Psychophysiology.

[17] Granholm EL, Panizzon MS, Elman JA, Jak AJ, Hauger RL, Bondi MW (2017). Pupillary responses as a biomarker of early risk for Alzheimer's disease. J Alzheimers Dis.

[18] Hampel H, Vergallo A, Perry G, Lista S, Alzheimer precision medicine I (2019). The Alzheimer Precision Medicine Initiative. J Alzheimers Dis.

[19] Hess EH, Polt JM (1964). Pupil size in relation to mental activity during simple problem-solving. Science.

[20] Just MA, Carpenter PA (1993). The intensity dimension of thought: pupillometric indices of sentence processing. Can J Exp Psychol.

[21] Kahneman D, Beatty J (1966). Pupil diameter and load on memory. Science.

[22] Karatekin C, Couperus JW, Marcus DJ (2004). Attention allocation in the dual-task paradigm as measured through behavioral and psychophysiological responses. Psychophysiology.

[23] Kawasaki A (1999). Physiology, assessment, and disorders of the pupil. Curr Opin Ophthalmol.

[24] Kelly SC, He B, Perez SE, Ginsberg SD, Mufson EJ, Counts SE (2017). Locus coeruleus cellular and molecular pathology during the progression of Alzheimer's disease. Acta Neuropathol Commun.

[25] Kucewicz MT, Dolezal J, Kremen V, Berry BM, Miller LR, Magee AL (2018). Pupil size reflects successful encoding and recall of memory in humans. Scientific Reports.

[26] Laeng B, Sirois S, Gredeback G (2012). Pupillometry: a window to the preconscious?. Perspect Psychol Sci.

[27] Lim JKH, Li Q-X, He Z, Vingrys AJ, Wong VHY, Currier N (2016). The eye as a biomarker for Alzheimer's disease. Front Neurosci.

[28] Marandi RZ, Gazerani P (2019). Aging and eye tracking: in the quest for objective biomarkers. Future Neurol.

[29] McKhann G, Knopman DS, Chertkow H, Hyman BT, Jack CR, Kawas CH (2011). The diagnosis of dementia due to Alzheimer's disease: recommendations from the National Institute on Aging-Alzheimer's Association workgroups on diagnostic guidelines for Alzheimer's disease. Alzheimers Dement.

[30] Ornek N, Dag E, Ornek K (2015). Corneal sensitivity and tear function in neurodegenerative diseases. Curr Eye Res.

[31] Peavler WS (1974). Pupil size, information overload, and performance differences. Psychophysiology.

[32] Piquado T, Isaacowitz D, Wingfield A (2010). Pupillometry as a measure of cognitive effort in younger and older adults. Psychophysiology.

[33] Prettyman R, Bitsios P, Szabadi E (1997). Altered pupillary size and darkness and light reflexes in Alzheimer's disease. J Neurol Neurosurg Psychiatry.

[34] Scinto LF, Frosch M, Wu CK, Daffner KR, Gedi N, Geula C (2001). Selective cell loss in Edinger-Westphal in asymptomatic elders and Alzheimer's patients. Neurobiol Aging.

[35] Scinto LF, Wu CK, Firla KM, Daffner KR, Saroff D, Geula C (1999). Focal pathology in the Edinger-Westphal nucleus explains pupillary hypersensitivity in Alzheimer's disease. Acta Neuropathol.

[36] Shen J, Wu J (2015). Nicotinic cholinergic mechanisms in Alzheimer's disease. Int Rev Neurobiol.

[37] Singh A, Verma S (2020). Use of ocular biomarkers as a potential tool for early diagnosis of Alzheimer’s disease. Indian J Ophthalmol.

[38] Sirois S, Brisson J (2014). Pupillometry. Wiley Interdiscip Rev Cogn Sci.

[39] Wahn B, Ferris DP, Hairston WD, Konig P (2016). Pupil sizes scale with attentional load and task experience in a multiple object tracking task. PLoS One.

[40] Wechsler D (1981). Wechsler Adult Intelligence Scale - Revised.

